# Graph Neural Network-Based Toxicity Prediction by Integrating Molecular Fingerprints and Knowledge Graph Features

**DOI:** 10.3390/toxics13110953

**Published:** 2025-11-05

**Authors:** Junjie Xie, Wei Liu, Wei Hu, Mei Ouyang, Tingting Huang

**Affiliations:** School of Informatics, Hunan University of Chinese Medicine, Changsha 410208, China; xjj@stu.hnucm.edu.cn (J.X.); huwei@hnucm.edu.cn (W.H.); mei_ouyang@hnucm.edu.cn (M.O.); 20233936@stu.hnucm.edu.cn (T.H.)

**Keywords:** molecular toxicity prediction, graph neural networks, knowledge graph, Tox21

## Abstract

Molecular toxicity prediction plays a crucial role in drug screening and environmental health risk assessment. Traditional toxicity prediction models primarily rely on molecular fingerprints and other structural features, while neglecting the complex biological mechanisms underlying compound toxicity, resulting in limited predictive accuracy, poor interpretability, and reduced generalizability. To address this challenge, this study proposes a novel molecular toxicity prediction framework that integrates knowledge graphs with Graph Neural Networks (GNNs). Specifically, we constructed a heterogeneous toxicological knowledge graph (ToxKG) based on ComptoxAI. ToxKG incorporates data from authoritative databases such as PubChem, Reactome, and ChEMBL, and covers multiple entities and relationships including chemicals, genes, signaling pathways, and bioassays. We then systematically evaluated six representative GNN models (GCN, GAT, R-GCN, HRAN, HGT, and GPS) on the Tox21 dataset. Experimental results demonstrate that heterogeneous graph models enriched with ToxKG information significantly outperform traditional models relying solely on structural features across multiple metrics including *AUC*, *F*1-score, *ACC*, and balanced accuracy (*BAC*). Notably, the GPS model achieved the highest *AUC* value (0.956) for key receptor tasks such as NR-AR, highlighting the critical role of biological mechanism information and heterogeneous graph structures in toxicity prediction. This study provides a promising pathway toward the development of interpretable and efficient intelligent models for toxicological risk assessment.

## 1. Introduction

Accurate assessment of chemical toxicity is essential for effectively avoiding and mitigating potential hazards of chemicals in areas such as drug screening [1], environmental risk evaluation, and public health management [2,3]. Traditional toxicity assessment methods mainly rely on in vivo animal experiments or in vitro cell assays, which are often time-consuming, costly, and raise ethical concerns. For example, evaluating the toxicity of a single compound can take several months or even years, with costs reaching millions of US dollars [4]. To address these challenges, computational toxicology has gradually emerged, aiming to develop rapid, efficient, and low-cost predictive models as alternatives to traditional experimental assessments [5].

Currently, methods widely applied to toxicity prediction tasks are primarily based on machine learning models of Quantitative Structure–Activity Relationship (QSAR), such as Support Vector Machines (SVM), Random Forests (RF), and Neural Networks (NN) [6]. These models typically perform toxicity classification by extracting molecular structural features or molecular fingerprints. Although these methods have improved assessment efficiency, their reliance on structural features while neglecting complex interactions between chemicals and biological systems results in insufficient predictive accuracy and a lack of clear mechanistic interpretation, thereby limiting their practical applications [7,8]. Existing studies have shown that in toxicity prediction tasks involving multiple mechanisms or complex biological pathways, the performance of traditional machine learning models still has room for improvement, with accuracy largely constrained by the representational capacity of molecular structural information [9,10].

In recent years, with the rapid advancement of artificial intelligence technologies, Graph Neural Networks (GNNs) have attracted increasing attention for their powerful representation learning capabilities in graph-structured data [11]. GNNs can effectively extract features from molecular graph structures and have been successfully applied to toxicity prediction, demonstrating considerable progress [12]. However, most existing GNN models primarily focus on structural feature learning, failing to capture the multi-scale and multi-level complex relationships between compounds and biological systems. This limitation restricts their generalizability and, in particular, their interpretability regarding toxicological mechanisms [13,14].

Meanwhile, Knowledge Graphs (KGs), as a structured knowledge representation approach [15], can effectively integrate heterogeneous biological information from multiple sources—including chemicals, genes, signaling pathways, and bioassays—thus providing richer semantic context and structured prior knowledge for mechanistic toxicology research [16,17]. Some recent studies have attempted to apply knowledge graphs to toxicity prediction, showing great potential in improving predictive performance and interpretability through graph embedding techniques [18]. Nevertheless, research that integrates knowledge graphs with GNNs for toxicity prediction remains limited. There is a particular lack of systematic methods for fusion of heterogeneous biological information (e.g., compound–gene–pathway associations) into GNN models. Furthermore, no prior study has explicitly and systematically evaluated the quantitative improvement in predictive performance and interpretability resulting from the incorporation of biological mechanism information.

To address these gaps, this study proposes a novel molecular toxicity prediction framework that integrates knowledge graphs with GNNs. Specifically, we constructed a toxicological knowledge graph (ToxKG) by extending the ComptoxAI knowledge graph [19], with data from publicly available databases, including PubChem, Reactome, and ChEMBL. ToxKG incorporates multiple entities such as chemicals, genes, pathways, and assays along with their complex relationships. We then combined heterogeneous features extracted from ToxKG, specifically compound–gene–pathway associations, with five classical molecular fingerprints (Atom-Pair, ECFP4, FP2, MACCS, and Morgan) as model inputs, and systematically evaluated six representative GNN models on the Toxicology in the 21st Century (Tox21) dataset. These models include Graph Convolutional Network (GCN) and Graph Attention Network (GAT) for homogeneous graphs, as well as Relational Graph Convolutional Network (R-GCN), Heterogeneous Representation Aggregation Network (HRAN), Heterogeneous Graph Transformer (HGT), and Graph Positioning System (GPS) for heterogeneous graphs. Experimental results show that incorporating knowledge graph information significantly improves predictive performance, with the GPS model achieving the highest *AUC* value of 0.956 on multiple receptor prediction tasks, outperforming traditional methods and substantially improving interpretability.

The objective of this study is to develop a toxicity prediction framework that integrates multi-scale structural features with semantic knowledge, thereby significantly improving both predictive accuracy and interpretability by incorporating heterogeneous associations among compounds, genes, and pathways. Ultimately, this research provides a new paradigm and methodological foundation for building mechanism-oriented intelligent models for toxicological prediction.

## 2. Materials and Methods

### 2.1. Dataset and Preprocessing

This study employed the publicly available Tox21 dataset for experimental evaluation. The Tox21 dataset was jointly developed by the United States Environmental Protection Agency (EPA, Washington, DC, USA) and the National Institutes of Health (NIH, Bethesda, MD, USA), and is widely used for multi-task classification studies of compound toxicity [20]. It contains activity assay results for 12 receptors.

To ensure data consistency and reliability, missing or uncertain experimental results were strictly filtered, compounds without definitive toxicity labels were marked as “−1” to explicitly distinguish missing values, and all samples lacking reliable toxicity annotations were excluded to ensure data consistency and reliability. After processing, a total of 7831 compounds with toxicity labels across 12 receptors were retained for subsequent modeling and analysis.

**Figure 1:**
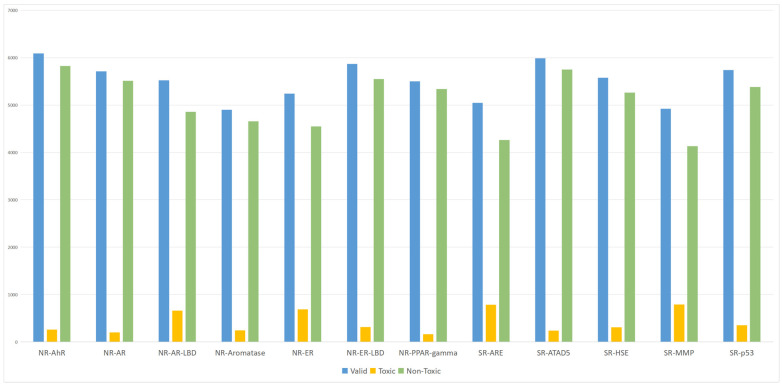
Statistical distribution of valid data, toxic compounds, and non-toxic compounds across 12 receptors in the Tox21 dataset.

The Simplified Molecular Input Line Entry System (SMILES) representations in the Tox21 dataset were used to retrieve corresponding PubChem CIDs via the PubChemPy toolkit. These CIDs were then cross-referenced with those recorded in the Toxicological Knowledge Graph (ToxKG), revealing that 6587 compounds were shared by both sources. After examining the completeness of their Compound–Gene–Pathway relationships in ToxKG, 6565 compounds were found to contain full relational information and were retained as the final dataset for all subsequent experiments. The list of these 6565 compounds used for model training and evaluation is provided in Supplementary Table S1. Both heterogeneous GNN models incorporating knowledge graph features and homogeneous GNN models using molecular fingerprints were trained on this unified dataset to ensure comparability and fairness across methods. Based on these data, we generated statistical plots (Figure 1) to illustrate the distribution of valid, toxic, and non-toxic compounds across the 12 receptors. As shown in Figure 1, a notable class imbalance exists between toxic and non-toxic compounds, which may cause the models to favor majority classes during training and consequently affect prediction performance.

To address this class imbalance issue, a reweighting strategy was introduced. Specifically, class weights were computed based on the proportion of each class, and higher loss weights were assigned to the minority class (toxic compounds), enabling the model to focus more on the predictive performance of underrepresented classes during training. This approach effectively alleviates the impact of data imbalance and enhances both the predictive performance and generalization ability of the model [21].

### 2.2. Construction of the Toxicological Knowledge Graph ToxKG

ToxKG was constructed by extending the publicly available ComptoxAI knowledge graph through a process of data filtering, cleaning, and supplementation, optimizing it for molecular toxicity prediction tasks. Firstly, the ontology data from ComptoxAI was imported into a Neo4j graph database. ComptoxAI serves as an integrative toxicological resource that aggregates data from multiple authoritative databases, comprising a diverse array of entities and relationships relevant to toxicology—including chemicals, genes, pathways, assays, key events, molecular initiating events, and adverse outcomes. Subsequently, PubChem was utilized to augment the structural information pertaining to chemical entities within ComptoxAI. This process involved standardizing chemical identifiers to PubChem CIDs, ensuring consistent and explicit structural representation for each chemical node, thereby facilitating more accurate feature extraction and downstream analysis. Furthermore, pathway-related information was expanded and enhanced through integration with the Reactome database. The hierarchical organization of biological pathways was systematically annotated to improve both the completeness and biological interpretability of pathway data within the graph. Additionally, compound–gene interaction data were enriched using ChEMBL. This integration reinforced existing associations between chemicals and genes, supplementing incomplete or absent interactions in the original ComptoxAI dataset, leading to a more comprehensive and reliable representation of toxicological mechanisms. Finally, the graph was refined by removing redundant and irrelevant relationships to enhance its structural conciseness and functional utility for subsequent analytical tasks.

After these processing steps, we obtained a heterogeneous ToxKG comprising multiple types of nodes and relationships. The specific entity and relationship types, along with their quantities, are shown in Table 1 and Table 2, respectively. The entity types in ToxKG include Chemical (19,446), Gene (17,517), Pathway (4558), as well as KeyEvent, AOP, MolecularInitiatingEvent, AdverseOutcome, and Assay. The relationship types include CHEMICALBINDSGENE, CHEMICALDECREASESEXPRESSION, CHEMICALINCREASESEXPRESSION, GENEINPATHWAY, and GENEINTERACTSWITHGENE, among others, all of which possess clear biological significance.

**Figure 2:**
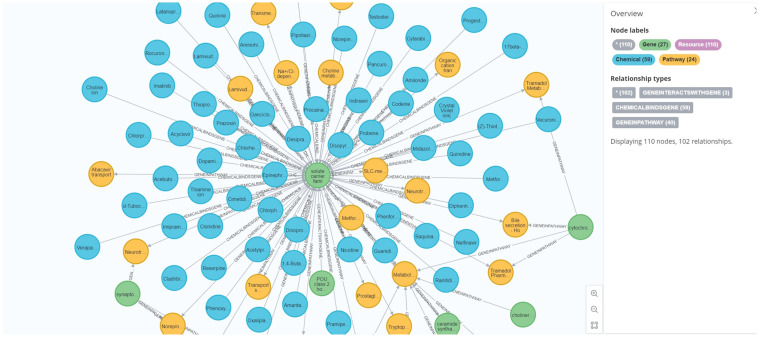
Example of a representative local subgraph in ToxKG.

In toxicity prediction experiments, this study primarily utilized three core node types—Chemical, Gene, and Pathway—along with their corresponding relationship information. These nodes and relationships were employed to construct the input data required for heterogeneous graph neural network models. Figure 2 illustrates a representative local subgraph from ToxKG, which demonstrates the complex and diverse biological relationships among compounds, genes, and signaling pathways, thereby providing richer and more interpretable biological feature inputs for the toxicity prediction task.

### 2.3. Construction of Node and Edge Feature Matrices

The toxicity prediction model constructed in this study integrates both molecular structural information and biological mechanism information. Accordingly, features were extracted from molecular structures and the toxicological knowledge graph, and then combined as input for the graph neural network.

First, for molecular structural feature extraction, five classical molecular fingerprints—MACCS, FP2, Morgan, Atom-Pair, and ECFP4—were selected. These fingerprints were computed for each compound using the RDKit. Since each fingerprint has distinct representational advantages, their concatenation enables a more comprehensive and accurate description of molecular structures, enhancing the robustness and generalization performance of toxicity prediction [22]. However, direct concatenation of these features results in high-dimensional vectors. To address this, Principal Component Analysis (PCA) was applied to reduce dimensionality, retaining 95% of the variance in the data, thus producing molecular node features of moderate dimensionality with reduced redundancy.

Second, with respect to the ToxKG, compound-associated gene and signaling pathway information was extracted, and graph embedding techniques were employed to transform graph-structured information into numerical vector representations. Specifically, eleven types of chemical–biological relationships in the graph (e.g., CHEMICALBINDSGENE, GENEINPATHWAY) were encoded as integer IDs or one-hot vectors, and their semantics were learned through the relation-encoding mechanisms of different GNN models. For example, in the GPS model, relation IDs are mapped to fixed-dimensional vectors via a learnable embedding layer, which are then combined with adjacent node features and input into the GINEConv module. In contrast, in models such as R-GCN, HGT, and HRAN, relation IDs are used to index relation-specific convolution weights, attention mappings, or attention matrices, thereby effectively distinguishing different semantic relationships during the modeling process. This approach enables the models to directly learn relational semantics in the toxicity prediction task without requiring additional pre-trained graph embeddings, thereby enhancing both feature relevance and model interpretability.

**Figure 3:**
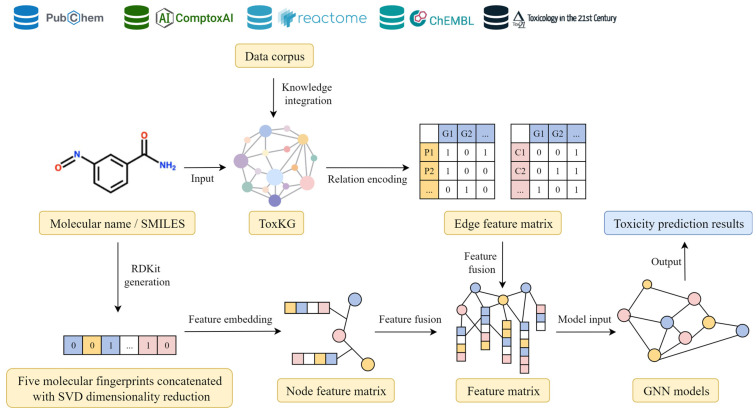
Overall experimental workflow.

Finally, the dimension-reduced molecular fingerprint-based node features, knowledge graph-derived node features, and relation-derived edge features were integrated to construct a unified node–edge feature matrix, which served as the input to the GNN models for subsequent toxicity prediction tasks. The integration process of molecular fingerprint features with graph structural information is illustrated in Figure 3, which demonstrates the overall workflow from multi-source data integration and feature extraction of molecular structures and graph information, to the construction of node and edge features and their final input into GNN models.

### 2.4. Construction and Training of Graph Neural Network Models

To systematically evaluate the performance improvement from knowledge graph integration in toxicity prediction tasks, six GNN models were selected for this study. These include two classical homogeneous graph models, GCN and GAT, as well as four models designed for heterogeneous graph structures: R-GCN, HRAN, HGT, and GPS.

GCN and GAT are widely used GNN models; however, they typically operate on homogeneous graph structures and thus cannot effectively distinguish between different types of relationships in heterogeneous knowledge graphs. Therefore, in this study, GCN and GAT were trained using only traditional molecular fingerprint features as inputs, without incorporating heterogeneous knowledge graph features. The experimental design of these two models primarily serves as a baseline to evaluate whether the integration of heterogeneous knowledge graph information significantly improves predictive performance.

In contrast, the four models—R-GCN, HRAN, HGT, and GPS—are inherently capable of handling heterogeneous graph structures. They can differentiate and utilize diverse node and edge features to integrate the complex biological information contained in the knowledge graph. Specifically, R-GCN employs relation-specific convolution mechanisms to distinguish between different types of edges; HRAN extends R-GCN by incorporating attention mechanisms over heterogeneous relations to enhance differential relational learning; HGT is built upon the Transformer architecture, explicitly modeling the heterogeneity of both node and edge types to better capture semantic interactions; and GPS combines the advantages of GNNs and Transformers, offering enhanced generalization ability and improved mechanistic interpretability.

For model training, this study was conducted under a unified training environment (GPU: NVIDIA RTX 3090 (NVIDIA Corporation, Santa Clara, CA, USA), CUDA 11.4, PyTorch Geometric (version 2.5.2)). The detailed parameter configurations are provided in Table 3. A five-fold cross-validation strategy was employed for model evaluation. In each fold, approximately 70% of the data were used as the training set and 20% as the validation set, while the remaining 10% were sequentially rotated as the test set, thereby ensuring the fairness and rigor of model assessment. All reported experimental results in this study are averaged metrics on the test sets across five folds, as the test set provides a more objective measure of the model’s generalization capability than the training or validation sets. The corresponding confusion matrices for each model during the training, cross-validation, validation, and test phases are provided in Supplementary Tables S2–S5.

### 2.5. Model Evaluation Metrics

In this study, different evaluation metrics were applied according to the model type.

For the homogeneous graph neural network models (GCN and GAT), three commonly used metrics (area under the receiver operating characteristic curve, *AUC*; balanced accuracy, *BAC*; and *F*1 score) were adopted to evaluate classification performance.

For the heterogeneous graph neural network models (R-GCN, HRAN, HGT, and GPS), five widely used metrics (*AUC*, *BAC*, overall accuracy (*ACC*), random accuracy (*RA*), and *F*1 score) were employed to comprehensively and objectively assess the predictive performance of the GNN models.

Among these metrics, the area under the Receiver Operating Characteristic curve (*AUC*) [23] is a classical metric extensively applied in classification tasks, and its calculation is given in Equation (1).

(1) $$\begin{array}{c} AUC=\frac{\sum_{i\in PositiveClass}\sum_{j\in NegativeClass}\;I(f(x_{i})>f(x_{j}))}{\left|PositiveClass|\times |NegativeClass\right|} \end{array}$$

Here, $f(x_{i})$ represents the prediction score of the model for a given sample, and I(⋅) denotes the indicator function, which takes the value of 1 when the condition inside the brackets is satisfied and 0 otherwise.

Balanced accuracy (*BAC*) [24] is particularly suitable for scenarios with imbalanced class distributions. It is defined as the average of sensitivity and specificity, as shown in Equation (2).

(2) $$\begin{array}{c} BAC=\frac{1}{2}\left(\frac{TP}{TP+FN}+\frac{TN}{TN+FP}\right) \end{array}$$

In these equations, *TP* denotes the number of true positives, *FN* the number of false negatives, *TN* the number of true negatives, and *FP* the number of false positives.

Overall accuracy (*ACC*) [25] measures the proportion of correctly predicted samples among all samples, and its definition is shown in Equation (3).

(3) $$\begin{array}{c} ACC=\frac{TP+TN}{TP+TN+FP+FN} \end{array}$$

Random accuracy (*RA*) [26,27] reflects the probability of a correct prediction by chance and is particularly useful as a baseline for comparison [28]. It can be computed based on the class distribution as shown in Equation (4).

(4) $$\begin{array}{c} RA=p_{pos}^{2}+p_{neg}^{2} \end{array}$$

Here $p_{pos}$ and $p_{neg}$ represent the proportions of positive and negative samples, respectively.

The *F*1 score is defined as the harmonic mean of *precision* and *recall* [29], making it suitable for evaluating classification performance under class imbalance. Its definition is given in Equation (5).

(5) $$\begin{array}{c} \;F1=\frac{2\times (Precision\times Recall)}{Precision+Recall},Precision=\frac{TP}{TP+FP},Recall=\frac{TP}{TP+FN} \end{array}$$

The above evaluation metrics assess the classification performance of the models from different perspectives, facilitating a comprehensive analysis of their performance differences in multi-label toxicity prediction tasks. In particular, balanced accuracy (*BAC*) is especially suitable for scenarios with imbalanced class distributions because it equally considers sensitivity and specificity, preventing the metric from being dominated by the majority class. This makes *BAC* a more robust indicator in toxicity datasets, where non-toxic samples often substantially outnumber toxic ones. Similarly, random accuracy (*RA*) provides a baseline reflecting the expected performance of a random classifier based on class proportions. Comparing the observed accuracy with *RA* allows for distinguishing genuine predictive capability from chance-level performance, ensuring that model evaluation remains fair and meaningful even under strong class imbalance.

### 2.6. Data, Code, and Model Availability Statement

The training code and model parameters of this study will be fully released on GitHub to facilitate replication or further research by other investigators. The corresponding code is available at: https://github.com/xiejunjie1010-sudo/Molecular-toxicity-prediction.git (accessed on 13 October 2025). The complete data and code can be accessed via the Hugging Face link: https://huggingface.co/xiejunjie/Molecular-toxicity-prediction (accessed on 13 October 2025).

### 2.7. AI Statement

We used ChatGPT (GPT-5, OpenAI, San Francisco, CA, USA) only for English language polishing to improve the clarity and readability of the manuscript. All ideas, analyses, interpretations, and conclusions were independently developed and verified by the authors.

## 3. Results

### 3.1. Toxicity Prediction Results of Baseline Models (GCN and GAT)

To evaluate the toxicity prediction performance of traditional graph neural network models without incorporating knowledge graph information, this study first constructed homogeneous graphs based on molecular fingerprints and employed the classical Graph Convolutional Network (GCN) and Graph Attention Network (GAT) as baseline models. Experiments were conducted on the 12 toxicity receptor tasks of the Tox21 dataset.

The experimental results (as shown in Table 4) reveal noticeable differences in predictive performance between the two baseline models across various toxicity tasks. Overall, the GCN model exhibits relatively stable performance across all metrics. In terms of *AUC*, it achieves higher predictive accuracy on SR-MMP (0.890), SR-ATAD5 (0.873), and NR-AhR (0.886), while performing relatively poorly on NR-ER (0.731) and NR-ER-LBD (0.805). The GCN model also attains the highest *BAC* value of 0.819 on NR-AR-LBD and a peak *F*1 score of 0.654 on SR-MMP, indicating its consistent ability to balance classification accuracy and precision-recall performance.

In comparison, the overall performance of the GAT model is slightly lower than that of GCN. Its best *AUC* values were observed in SR-MMP (0.883) and NR-AhR (0.874), while the highest *BAC* and *F*1 scores appeared in SR-MMP (0.797) and NR-AhR (0.652), respectively. These results suggest that although GAT can capture certain attention-based dependencies, it does not significantly outperform GCN in the toxicity prediction of the Tox21 dataset, especially in maintaining a balance between accuracy and recall.

### 3.2. Experimental Results of Heterogeneous Graph Models Incorporating Knowledge Graphs

To further enhance the accuracy and generalizability of molecular toxicity prediction, the constructed ToxKG was integrated into the graph neural network models. Four heterogeneous graph models—R-GCN, HRAN, HGT, and GPS—were employed for experimental evaluation. To comprehensively assess model performance, five evaluation metrics were adopted: *ACC*, *RA*, *BAC*, *F*1 score, and *AUC*. The experimental results, as shown in Table 5, Table 6, Table 7 and Table 8, present the performance of the four models across the 12 receptor tasks of the Tox21 dataset. The detailed results for each fold of the test sets and their corresponding averaged values for the GPS, HGT, HRAN, and R-GCN models can be found in Supplementary Tables S7–S9.

The results indicate that the GPS model achieved the best overall performance, attaining the highest *AUC* value (0.956) on the NR-AR receptor task and maintaining high *ACC* and *BAC* values across most receptor tasks, demonstrating its strong predictive capability and generalization performance. The R-GCN model also exhibited outstanding results, achieving the highest *AUC* value (0.966) on the SR-p53 receptor task, reflecting its superior feature extraction and task adaptability. The HGT model showed stable performance on tasks such as NR-AR-LBD and SR-ARE, with *AUC* values of 0.941 and 0.937, respectively, indicating its robustness in modeling multi-source heterogeneous information. Although the HRAN model showed slightly lower overall performance, it still outperformed the homogeneous baseline models in receptor tasks such as NR-AR and NR-ER-LBD.

A comprehensive analysis of the five metrics (*BAC*, *F*1, *AUC*, *ACC*, and *RA*) reveals that heterogeneous graph models incorporating the ToxKG knowledge graph significantly improved toxicity prediction performance. The *AUC* values of all models generally exceeded 0.90, with several key receptor tasks achieving remarkable results. These findings demonstrate that heterogeneous graph neural networks integrating toxicological knowledge graphs possess clear advantages in molecular toxicity prediction, particularly in addressing data imbalance and complex toxicological mechanisms.

### 3.3. Comparison and Contrast Between Heterogeneous Models and Baseline Models

To further evaluate the practical improvement in toxicity prediction performance brought by incorporating knowledge graphs, a systematic comparative analysis was conducted between the baseline models without graph information (GCN and GAT) and the heterogeneous graph models incorporating the ToxKG knowledge graph (R-GCN, HRAN, HGT, and GPS).

A comprehensive comparison of the experimental results in Table 4, Table 5 and Table 9 reveals that heterogeneous graph models enriched with knowledge graph information consistently outperformed the baseline models in most receptor tasks, particularly in terms of *AUC*, which reflects the overall predictive capability of the models. For example, the GPS model achieved *AUC* values of 0.954 and 0.924 on the SR-MMP and SR-p53 receptor tasks, respectively, representing improvements of more than 0.06 to 0.09 compared with the baseline models (GCN: 0.890 and 0.839; GAT: 0.883 and 0.826). Similarly, for the NR-AR receptor task, the GPS model achieved an *AUC* of 0.956, whereas GCN and GAT yielded only 0.779 and 0.781, respectively, with improvements exceeding 0.17.

In addition, the *BAC* and *F*1 metrics of heterogeneous graph models also exhibited consistent improvement. These results indicate that incorporating heterogeneous information that captures biological mechanisms and molecular interactions effectively compensates for the limitations of traditional homogeneous graph models that rely solely on molecular structural information. This significantly enhances model performance and generalization in complex toxicity prediction tasks. Collectively, these experimental findings further validate the importance and effectiveness of the proposed knowledge graph integration strategy for molecular toxicity prediction.

### 3.4. Comparative Analysis with Other Tox21-Related Research

To further validate the effectiveness of the proposed model, we compared it against several representative methods from recent studies on Tox21 toxicity prediction. These included the random forest model from the MoleculeNet project (Wu et al., 2018) [30], the molecular similarity–naïve Bayes model (MS-NB) proposed by Drwal et al. (2015) [31], the deep learning model DeepTox, which won the Tox21 Challenge (Mayr et al., 2016) [32], and the random forest model with 5-fold cross-validation (RF-CV5) reported by Capuzzi et al. (2016) [33]. In addition, we incorporated two advanced graph neural network frameworks, namely the equivariant geometric model ET (Cremer et al., 2023) [34] and the few-shot multi-task model JLGCN-MTT (Zhao et al., 2025) [35]. The *AUC* values across all 12 receptor tasks in the Tox21 dataset are summarized in Table 9. The complete experimental data of the best-performing GPS model and its corresponding confusion matrices are provided in Supplementary Tables S10 and S11, respectively.

The comparison results indicate that the proposed GPS model outperforms existing mainstream methods in most receptor tasks. For instance, in critical toxicity endpoints such as NR-AR, NR-AR-LBD, SR-ATAD5, and SR-MMP, the GPS model achieved *AUC* values of 0.956, 0.937, 0.941, and 0.954, respectively, which are substantially higher than those of traditional random forest (0.847, 0.775, 0.731, 0.927), MS-NB (0.83, 0.89, 0.80, 0.90), and RF-CV5 (0.82, 0.91, 0.83, 0.92). The DeepTox model achieved an average *AUC* of 0.853 on the Tox21 final blind test set, demonstrating strong generalization ability; however, the proposed GPS model still achieved a higher average *AUC* of 0.911 under cross-validation, reflecting more consistent performance across receptor tasks.

Since most Tox21-related studies have adopted *AUC* as the primary evaluation metric, other measures such as *F*1, *BAC*, *ACC*, and *RA* are not comprehensively reported in the literature. Therefore, *AUC* served as the main basis for comparison in this section. These results further confirm the advancement and robustness of the proposed heterogeneous GNN model integrating knowledge graph information for molecular toxicity prediction.

### 3.5. Experimental Conclusions and Future Perspectives

The proposed heterogeneous graph neural network model, which incorporates knowledge graphs, demonstrated significantly superior performance compared with traditional homogeneous graph baselines in molecular toxicity prediction tasks on the Tox21 dataset. When evaluated against both classical machine learning models and recent representative GNN approaches, the proposed model achieved leading *AUC* values across the majority of toxicity endpoints. These experimental results strongly suggest that deep integrating mechanistic biological information with molecular structural features is a pivotal strategy for improving the accuracy and generalizability of molecular toxicity prediction.

Future work will further expand and refine the entity and relationship types within the toxicological knowledge graph, thereby enriching the model’s capacity to capture complex biological mechanisms. In addition, multi-modal molecular representation approaches—such as incorporating three-dimensional structures, molecular images, and textual information—will be explored to further enhance the generalization and interpretability of the model. These advancements are expected to facilitate the practical application of intelligent platforms for molecular toxicity prediction.

## 4. Discussion

This study introduces a pioneering approach to molecular toxicity prediction by systematically integrating the Toxicological Knowledge Graph (ToxKG) with multiple graph neural network models. The experimental results demonstrate that incorporating biological mechanism information from knowledge graphs—including compounds, genes, and pathways—into GNN models can significantly enhance prediction accuracy, model robustness, and generalizability for complex biological responses.

Traditional molecular toxicity prediction methods, such as random forests, support vector machines, and classical QSAR models, primarily rely on chemical structural features or molecular fingerprints. These approaches are limited in capturing multi-level regulatory relationships among compounds, genes, and pathways, which restricts their capacity to model complex toxicological responses and weakens their interpretability and translational value [32]. By integrating data from ComptoxAI, PubChem, Reactome, and ChEMBL, this study constructed ToxKG, a multi-layered toxicological knowledge graph incorporating compounds, genes, signaling pathways, and assay labels, thereby providing heterogeneous relational information that reflects real biological processes.

At the biological level, many toxicity endpoints (e.g., hepatotoxicity, genotoxicity) are not determined by molecular structure alone but depend on the synergistic effects of compounds with multiple genes and signaling pathways [36,37]. For example, certain compounds can regulate NR family receptors, alter downstream gene expression, and modulate metabolic pathways, ultimately producing specific toxicological effects [38]. The knowledge graph structure explicitly encodes such complex causal relationships and regulatory networks into molecular representations. Heterogeneous GNNs, through multi-type nodes and relations, not only enrich the feature space of compounds but also enhance the capacity of models to capture and express mechanistic information. This enables the models to automatically learn [39] which genes and pathways play central roles in specific toxicological responses, thereby achieving higher predictive accuracy and stronger generalization ability [40,41].

This study systematically compared the performance of homogeneous models (GCN, GAT) with heterogeneous models (R-GCN, HRAN, HGT, GPS) on the Tox21 dataset. The experiments showed that heterogeneous models, particularly GPS and R-GCN, substantially outperformed the baselines across multiple metrics including *AUC*, *F*1, and *BAC*. The GPS model achieved the most significant *AUC* improvements in endpoints such as NR-AR and SR-MMP, while R-GCN demonstrated superior performance on SR-p53, highlighting the effectiveness of incorporating heterogeneous relations in enhancing the representation of toxicological mechanisms. These findings are consistent with recent trends in leveraging graph embeddings and semi-supervised learning strategies to improve toxicity prediction, further emphasizing the importance of integrating multi-source heterogeneous information in this task [42].

## 5. Conclusions

Although this study has achieved significant progress on the twelve receptor endpoints of the Tox21 dataset, several limitations remain. First, the generalization ability of the proposed models still needs to be validated on more diverse real-world toxicity datasets. Second, the class imbalance issue inherent in Tox21 continues to hinder performance, and the prediction accuracy for minority classes requires further improvement. Future work may incorporate few-shot learning and semi-supervised approaches, combined with class weighting and synthetic sample generation strategies, to enhance the prediction of low-frequency toxic responses. In addition, the ToxKG can be further expanded by including additional biological entities such as protein isoforms, cell types, and disease nodes, as well as by developing node- and edge-level attention-based interpretability tools to uncover key biological mechanisms. Furthermore, future research will explore the establishment of an applicability domain (AD) [43] to quantitatively define the reliable chemical space of model predictions and integrate conformal prediction (CP) [44,45] techniques to provide statistically valid confidence levels for each toxicity prediction, thereby enhancing both the interpretability and reliability of the proposed framework. Future research will also focus on extending the applicability of the framework by evaluating its performance on multiple toxicity endpoints and large-scale compound libraries, improving interpretability through attention mechanisms and causal reasoning to identify critical genes, pathways, and regulatory relationships, and advancing multimodal knowledge graph integration by incorporating three-dimensional molecular structures, molecular images, and literature-derived information to further enhance predictive power and practical utility. In summary, this study proposed and validated a novel framework that integrates toxicological knowledge graphs with graph neural networks for molecular toxicity prediction, significantly improving predictive accuracy, generalization ability, and mechanistic interpretability. These findings highlight the theoretical significance and practical potential of the proposed framework in environmental toxicology and drug safety evaluation.

## Figures and Tables

**Table 1:** Statistical summary of entity types in ToxKG.

Entity Types	Count
Chemical	19,446
Gene	17,517
Pathway	4558
KeyEvent	1111
AOP	262
MolecularInitiatingEvent	193
AdverseOutcome	156
Assay	59

**Table 2:** Statistical summary of relation types in ToxKG.

Relation Types	Count
CHEMICALHASINACTIVEASSAY	856,330
CHEMICALDECREASESEXPRESSION	21,047
CHEMICALBINDSGENE	11,525
CHEMICALHASACTIVEASSAY	65,618
CHEMICALINCREASESEXPRESSION	18,710
GENEINPATHWAY	179,464
GENEINTERACTSWITHGENE	147,008
AOPINCLUDESKE	1810
KEINCLUDEDINAOP	1810
KEYEVENTTRIGGERS	1338
KEYEVENTTRIGGEREDBY	239

**Table 3:** Parameter configuration of the models.

Parameter Configuration	Specific Setting
Hidden layer dimension	256
Activation function	LeakyReLU
Dropout ratio	0.3
Optimizer	Adam
Initial learning rate	0.001
Training strategy	Five-fold cross-validation
GPU environment	NVIDIA RTX 3090
Deep learning framework	PyTorch Geometric

**Table 4:** Comparison of experimental results between GCN and GAT. All results in this table represent the averaged test-set results obtained from five-fold cross-validation.

Receptor	GCN_*BAC*	GCN_*F*1	GCN_*AUC*	GAT_*BAC*	GAT_*F*1	GAT_*AUC*
NR-AR	0.752	0.602	0.779	0.765	0.595	0.874
NR-AR-LBD	**0.819**	0.69	0.858	0.752	0.589	0.781
NR-AhR	0.751	0.602	**0.886**	0.82	0.652	0.862
NR-Aromatase	0.664	0.421	0.818	0.71	0.457	0.798
NR-ER	0.654	0.431	0.731	0.658	0.428	0.724
NR-ER-LBD	0.737	0.549	0.805	0.722	0.512	0.797
NR-PPAR-γ	0.654	0.383	0.8	0.699	0.437	0.814
SR-ARE	0.706	0.519	0.813	0.681	0.477	0.784
SR-ATAD5	0.653	0.406	0.873	0.688	0.447	0.858
SR-HSE	0.667	0.43	0.769	0.673	0.442	0.771
SR-MMP	0.777	**0.654**	0.89	**0.797**	**0.672**	**0.883**
SR-p53	0.697	0.473	0.839	0.716	0.497	0.826

**Table 5:** Performance of the GPS model across 12 Tox21 receptors. All results in this table represent the averaged test-set results obtained from five-fold cross-validation.

Receptor	*ACC*	*RA*	*BAC*	*F*1	*AUC*
NR-AR	**0.977**	0.932	0.87	0.72	**0.956**
NR-AR-LBD	0.906	0.789	0.868	0.746	0.937
NR-AhR	0.960	0.918	0.793	0.599	0.883
NR-Aromatase	0.950	0.906	0.779	0.54	0.912
NR-ER	0.862	0.771	0.802	0.626	0.87
NR-ER-LBD	0.947	0.898	0.793	0.579	0.885
NR-PPAR-γ	0.969	0.942	0.756	0.48	0.881
SR-ARE	0.886	0.737	0.850	0.683	0.914
SR-ATAD5	0.963	0.924	0.803	0.558	0.941
SR-HSE	0.944	0.894	0.778	0.564	0.874
SR-MMP	0.911	0.730	**0.885**	**0.776**	0.954
SR-p53	0.951	0.884	0.806	0.589	0.924

**Table 6:** Performance of the HGT model across 12 Tox21 receptors. All results in this table represent the averaged test-set results obtained from five-fold cross-validation.

Receptor	*ACC*	*RA*	*BAC*	*F*1	*AUC*
NR-AR	**0.97**	0.932	0.83	0.57	0.877
NR-AR-LBD	0.912	0.789	**0.89**	0.705	0.941
NR-AhR	0.96	0.917	0.869	0.614	0.895
NR-Aromatase	0.924	0.906	0.828	0.484	0.895
NR-ER	0.873	0.771	0.822	0.585	0.855
NR-ER-LBD	0.937	0.898	0.859	0.579	0.905
NR-PPAR-γ	0.96	0.942	0.811	0.519	0.921
SR-ARE	0.911	0.737	0.895	**0.738**	0.937
SR-ATAD5	0.949	0.924	0.88	0.575	**0.946**
SR-HSE	0.929	0.894	0.795	0.49	0.85
SR-MMP	0.903	0.731	0.878	0.71	0.929
SR-p53	0.944	0.884	0.856	0.61	0.944

**Table 7:** Performance of the HRAN model across 12 Tox21 receptors. All results in this table represent the averaged test-set results obtained from five-fold cross-validation.

Receptor	*ACC*	*RA*	*BAC*	*F*1	*AUC*
NR-AR	**0.976**	0.932	0.834	0.693	0.907
NR-AR-LBD	0.925	0.789	0.829	0.704	0.897
NR-AhR	0.973	0.917	0.815	0.669	0.874
NR-Aromatase	0.943	0.906	0.726	0.473	0.82
NR-ER	0.892	0.771	0.792	0.643	0.845
NR-ER-LBD	0.952	0.898	0.769	0.604	0.879
NR-PPAR-γ	**0.976**	0.942	0.735	0.516	0.869
SR-ARE	0.903	0.737	**0.841**	**0.729**	0.912
SR-ATAD5	0.962	0.924	0.71	0.447	0.865
SR-HSE	0.951	0.894	0.739	0.52	0.851
SR-MMP	0.916	0.73	0.832	0.724	**0.916**
SR-p53	0.954	0.884	0.788	0.618	0.888

**Table 8:** Performance of the R-GCN model across 12 Tox21 receptors. All results in this table represent the averaged test-set results obtained from five-fold cross-validation.

Receptor	*ACC*	*RA*	*BAC*	*F*1	*AUC*
NR-AR	**0.983**	0.932	0.835	0.719	0.913
NR-AR-LBD	0.941	0.789	0.842	0.743	0.937
NR-AhR	0.979	0.917	0.822	0.722	0.909
NR-Aromatase	0.962	0.906	0.753	0.582	0.938
NR-ER	0.92	0.771	0.797	0.672	0.855
NR-ER-LBD	0.967	0.898	0.787	0.657	0.889
NR-PPAR-γ	0.981	0.942	0.8	0.674	0.944
SR-ARE	0.933	0.737	0.858	0.78	0.941
SR-ATAD5	0.971	0.924	0.76	0.597	0.953
SR-HSE	0.965	0.894	0.786	0.629	0.899
SR-MMP	0.934	0.730	**0.867**	**0.788**	0.955
SR-p53	0.966	0.884	0.828	0.707	**0.966**

**Table 9:** Comparative results of *AUC*. The *AUC* values of the ET and MS-NB methods were approximated from the bar charts in the original papers and may contain small deviations.

Receptor	RF-MN	ET	MS-NB	DeepTox	RF-CV5	JLGCN-MTT	GPS
NR-AhR	0.879	0.75	0.90	**0.928**	0.91	0.894	0.883
NR-AR	0.847	0.80	0.83	0.807	0.82	0.905	**0.956**
NR-AR-LBD	0.775	0.90	0.89	0.879	0.91	0.893	**0.937**
NR-Aromatase	0.773	0.78	0.82	0.834	0.82	0.907	**0.912**
NR-ER	0.737	0.65	0.80	0.810	0.79	**0.917**	0.870
NR-ER-LBD	0.778	0.65	0.88	0.814	0.86	**0.889**	0.885
NR-PPAR-γ	0.878	0.76	0.82	0.861	0.81	**0.945**	0.881
SR-ARE	0.846	0.82	0.81	0.840	0.83	0.883	**0.914**
SR-ATAD5	0.731	0.80	0.80	0.793	0.83	0.899	**0.941**
SR-HSE	0.732	0.85	0.87	0.865	0.80	**0.900**	0.874
SR-MMP	0.927	0.88	0.90	0.942	0.92	0.940	**0.954**
SR-p53	0.724	0.83	0.84	0.862	0.82	0.854	**0.924**
Average *AUC*	0.803	0.831	0.847	0.853	0.84	0.894	**0.911**

## Data Availability

The datasets and code supporting this study are openly available at GitHub and Hugging Face. Key curated tables are also provided in the Supplementary Materials (Tables S1–S11).

## Supplementary Materials

The following supporting information can be downloaded at: https://www.mdpi.com/article/10.3390/toxics13110953/s1, Table S1: List of 6565 compounds used for model training and evaluation, including toxicity labels across 12 receptors, SMILES, and PubChem CIDs. Table S2: GPS model: confusion matrices for training/validation/test sets in each fold of five-fold cross-validation on the Tox21 dataset. Table S3: HGT model: confusion matrices for training/validation/test sets in each fold of five-fold cross-validation on the Tox21 dataset. Table S4: HRAN model: confusion matrices for training/validation/test sets in each fold of five-fold cross-validation on the Tox21 dataset. Table S5: R-GCN model: confusion matrices for training/validation/test sets in each fold of five-fold cross-validation on the Tox21 dataset. Table S6: GPS model: per-fold test-set results and the averaged results over five folds. Table S7: HGT model: per-fold test-set results and the averaged results over five folds. Table S8: HRAN model: per-fold test-set results and the averaged results over five folds. Table S9: R-GCN model: per-fold test-set results and the averaged results over five folds. Table S10: Complete experimental data of the best-performing model (GPS) on the Tox21 dataset. Table S11: The best experimental results observed in this study and their corresponding confusion matrices.
